# Geriatric Assessment and Management, Prehabilitation and Rehabilitation for Older Aldults with Non-Colorectal Digestive Cancers

**DOI:** 10.3390/cancers17091589

**Published:** 2025-05-07

**Authors:** Amélie Aregui, Janina Estrada, Madeleine Lefèvre, Anna Carteaux-Taieb, Geoffroy Beraud-Chaulet, Pascal Hammel, Virginie Fossey-Diaz, Thomas Aparicio

**Affiliations:** 1 Paris Nord Oncogeriatrics Coordination Unit, Bretonneau and Saint Louis Hospitals, AP-HP, 75018 Paris, France; 2 Geriatric Out-Patient Unit, Bretonneau Hospital, AP-HP, 75018 Paris, France; 3 Department of Digestive and Endocrine Surgery, Saint-Louis Hospital, AP-HP, 75010 Paris, France; 4 Geriatrics Departement, Paul Brousse Hospital, AP-HP, 94800 Villejuif, France; 5 Oncogériatric Department, Gustave Roussy Institut, 94800 Villejuif, France; 6 Medical Oncology Department, Paul Brousse Hospital, AP-HP, Université Paris Saclay, 94800 Villejuif, France; 7 Geriatrics Departement, Bretonneau Hospital, AP-HP, 75018 Paris, France; 8 Gastroenterology Department, Saint Louis Hospital, AP-HP, Université Paris Cité, 75010 Paris, France

**Keywords:** geriatric comprehensive assessment, non-colorectal digestive cancer, older adults, treatment tolerance, quality of life

## Abstract

Background: The incidence of cancer in older patients is high, reaching 2.3 million world-wide in 2018 for patients aged over 80. Because the characteristics of this population make therapeutic choices difficult, co-management between geriatricians and other cancer specialists has gradually become essential. Methods: This narrative review aims to synthesize current data on the contribution of geriatric assessment in the management of elderly patients with non-colorectal digestive cancers. Oncogeriatric assessment is multi-domain, including the evaluation of co-morbidities, autonomy, nutrition, cognition, mood, and functional assessment. Results: Oncogeriatric parameters are predictive of mortality and adverse events. In the peri-operative phase of non-colorectal digestive cancer surgical management, geriatric management can assist in the decision-making process, identify frailties, and arrange a specific and personalized trimodal preoperative rehabilitation program, including nutritional management, adapted physical activity, and psychological care. Its aim is to limit the risks of confusion and of decompensation of comorbidities, mainly cardio-respiratory, which is associated with the highest morbidity in biliary-pancreatic surgery for older adults, facilitate recovery of previous autonomy when possible, and shorten hospital stay. For metastatic cancers, or during multimodal management, such as peri-operative chemotherapy for localized gastric cancers or pre-operative radio-chemotherapy for oesophageal or rectal cancers, specific assessment of the tolerance of chemotherapy is necessary. Neuropathic toxicity and chemobrain have a greater impact on elderly patients, with an increased loss of autonomy. Joint geriatric management can reduce the rate of grade 3–5 adverse effects of chemotherapy in particular and improve quality of life. Conclusions: Co-management between geriatricians and other specialties should be encouraged wherever possible.

## Simple Summary

Digestive cancers are common among older adults, yet their management can be more challenging in this population given the physiological differences between older and younger patients. That is why geriatricians now work with cancer specialists to help in the decision process and identify patient frailties. Through a global assessment, including lifestyle, muscle function, nutrition, and psychological well-being, geriatricians can help build a tailored treatment. This may involve, depending on the chosen treatments, preparing patients for the surgery and improving their postoperative recovery, or mitigating the side effects of chemotherapy and other therapies. However, geriatrician contributions in the management of non-colorectal cancers (oesophagus, stomach, liver, pancreas, or biliary tract cancers) is not as well-known as in colon or rectal cancer. This paper aims to summarize current research on the geriatric management of non-colorectal digestive cancers: how it can be done and how it can improve treatment outcomes and patients’ quality of life.

## 1. Introduction

In 2018, 13.3% of newly diagnosed cancer cases involved patients aged 80 and over, amounting to 2.3 million new cases globally across all cancer types. Given the demographic shift towards an aging population and the heightened incidence of cancer with advancing age, this statistic is anticipated to escalate to 6.9 million by 2050, constituting 21.5% of newly diagnosed cancers [1]. The importance of non-colorectal digestive cancers is far from negligible, since stomach, liver, and oesophageal cancers are among the 10 most frequently diagnosed cancers worldwide, and stomach, liver, oesophageal, and pancreatic cancers are among the top 10 leading causes of cancer deaths [2]. The aging process is associated with a decline in 10-year net survival, the observed survival if cancer is the only cause of death, highlighting the need for further investigation. Multiple factors likely contribute to this decline, including delayed diagnosis in older patients and limitations imposed by comorbidities. For instance, constraints on diagnostic explorations such as contrast injections in cases of renal failure or general anaesthesia before endoscopic ultrasound guided fine needle biopsy in pancreatic cancer may play a role [3]. Cognitive impairment, prevalent in the geriatric population, further disrupts the continuum of care, leading to missed appointments and lapses in follow-up.

Optimal treatment selection poses another challenge. The SAGE Prospective Multicenter Cohort survey revealed an underrepresentation of older patients in therapeutic trials focused on colorectal cancer [4]. This underrepresentation extends to non-colorectal cancers, complicating the decision-making process. Given the inherent heterogeneity of this population, some robust individuals may receive treatments similar to that of younger patients, while more fragile patients may require tailored interventions. Given the generally poor prognosis of non-colorectal digestive cancers (accounting for 26% of cancer-related deaths [2]), and even lower among older adults, we have chosen to search the literature for the particularities of this population and any specific management. Considering the small number of patients in this sub-population in the literature, and the fact that the same specialist manages each cancer, we decided to group them together. Unlike colorectal cancers, the lack of specific recommendations for non-colorectal digestive cancer in older adults adds to the complexity of treatment decision making [5,6].

Consequently, navigating through this intricate decision-making process is crucial, aiming to provide the best possible care for the patient without undertreating them, while preserving autonomy and quality of life. Given these unique challenges, collaboration between geriatricians and other specialized healthcare professionals has gradually become mandatory.

This narrative review aims to describe geriatric assessment and its contribution to the management of older adults with non-colorectal digestive cancers.

## 2. Methods

A literature search was conducted on PubMed and Google Scholar, encompassing English-language articles published between January 2000 and 2024. The references in the identified articles were also reviewed to find additional publications of interest.

The keywords used were geriatric assessment, elderly, older patients, digestive cancer, hepatobiliary-pancreatic cancer, oesophagogastric cancer, prehabilitation, rehabilitation, fast-track surgery, systemic treatments, chemotherapy, cognitive disorders.

Articles were included in the review based on relevancy and publication types.

## 3. Oncogeriatric Evaluation Specific to the Non-Colorectal Digestive Cancers, Decision Support

### 3.1. Identifying Frail Patients

While the ECOG PS (PS adapted to the older adults, mainly by adapting references to the ability to work) scale offers a comprehensive assessment of general condition, its applicability for older patients is not universal, and it lacks sensitivity in predicting loss of autonomy [5].

For individuals above 70 years, the International Society of Geriatric Oncology (SIOG) recommends utilizing the G8 score, validated by the Oncodage study [6], and advocates for an in-depth geriatric assessment when the score is less than or equal to 14. The modified G8, although more specific and capable of targeting patients with abnormal geriatric assessments, particularly by incorporating considerations of heart disease, which are crucial for pancreatic and hepatobiliary surgery, is underused in routine clinical practice [7].

### 3.2. Comprehensive Geriatric Assessment (CGA)

The CGA aims to identify pivotal factors and comorbidities influencing the balance between curative and palliative care. This in-depth evaluation encompasses the patient’s overall condition, autonomy, nutritional, cognitive, and thymic status, along with an assessment of comorbidities, functional abilities, and the social environment [8]. The geriatrician uses different scales for each of these domains during a lengthy consultation. Geriatricians have access to multiple assessment scales, leading to a lack of standardization in geriatric data collection for clinical trials. This variability contributes to the absence of specific recommendations for non-colorectal digestive cancer management in older adults. To resolve the matter, tools such as the Geriatric Core Dataset have been developed, through a Delphi consensus process, aiming to standardize geriatric data collection in clinical trials and facilitate comparisons across studies [9].

#### 3.2.1. Loss of Autonomy and the Role of the Caregiver

Autonomy, which demonstrates significant heterogeneity within this population, is evaluated according to SIOG recommendations through the Activity of Daily Living (ADL, six items: washing, dressing, walking, toileting, eating, continence evaluation) and the Instrumental Activity of Daily Living (IADL, eight items: ability to use telephone, shopping, food preparation, housekeeping, laundry, mode of transportation, responsibility for own medications, ability to handle finances). Patients are considered dependent if they require assistance with at least one of these items, with IADLs being more sensitive and displaying earlier impacts. They appear to predict treatment feasibility and chemotherapy toxicity [10]. Impaired ADL and IADL are also associated with unscheduled hospitalization and early three-month mortality [11].

After open digestive, hepatobiliary, or gastrointestinal surgery, the decline in ADL is evident irrespective of age, with a more pronounced decline after the age of 80 [12].

The geriatrician, when assessing autonomy, also explores available resources such as the presence of a caregiver at home or nearby, which influences the patient’s quality of life [13]. This aspect is crucial, especially in cases of memory disorders, for detecting adverse effects of systemic treatments and ensuring therapy adherence. The Zarit scale should be employed to assess caregiver burden, aiming to mitigate its impact on the caregiver’s health [14].

In case of absence of a caregiver or in the event of caregiver exhaustion, it is essential to consider the introduction of professional help at the home.

#### 3.2.2. Malnutrition

Malnutrition is intricately linked to cancer, inflammation, hyper-catabolism, digestive tract damage, and co-morbidities. The 2023 Nutriage study revealed that approximately half of the patients referred to a oncogeriatrician had lost more than 5% of their body weight in the last 6 months [15]. In particular, the prevalence of malnutrition in oesogastric and digestive cancers among a population with a median age of 63 is approximately 55% (pancreas 54%, liver 55%, oesophagogastric 53%) [16,17].

The prevalence of malnutrition increases with age [18] and is higher in digestive cancers. In the study by Poisson et al., involving patients referred to an oncogeriatrician, mortality at 6 months was 20.5%, and cachexia increased the risk of mortality independently of age. Its prevalence is estimated at 52% [19]. In a 2021 review, researchers found a significant link between nutritional status and higher intermediate- and long-term mortality [20].

Symptoms associated with undernutrition differ somewhat in older patients, with loss of appetite, difficulty chewing, dry mouth, and fatigue, but less nausea and vomiting than in younger patients [18,21]. Following weight loss, regaining their initial weight is harder for older individuals than their younger counterpart, even over the long term [22]. Managing malnutrition in older adults can be challenging, but evidence shows that oral nutritional supplements help prevent weight loss, even in pancreatic cancer patients [23]. Measuring resting energy expenditure could aid in the selection of personalized treatment, considering that cancer is associated with dysfunctions in energy homeostasis. An increase in resting energy expenditure can lead to weight loss and is associated with a higher risk of early chemotherapy-limiting toxicity in older cancer patients [24].

#### 3.2.3. Walking and Muscle Function

Changes in body composition associated with aging, characterized by increased fat mass and diminished lean mass, coupled with weight loss and muscle mass reduction, contribute to impaired walking, compromised balance, and a decline in autonomy. Current recommendations for nutritional assessment by oncogeriatricians emphasize the investigation of sarcopenia. Sarcopenia is linked to a 6-month mortality rate of 23% in an all-cancer population averaging 83 years of age (comprising 10.2% non-colorectal digestive cancers and 15% colorectal cancers) [15]. In cancers of the oesophagogastric junction, sarcopenia becomes more prevalent as patients age. Its persistence even after intensive renutrition during neoadjuvant chemotherapy is correlated with heightened mortality and could be considered as a criterion for determining the appropriateness of surgical management [25].

Beyond muscle mass, muscle density may also influence functionality. Walking speed has been correlated with mortality after the age of 65 and is associated with frailty, with a threshold of 1 m per second (m/s) for older cancer patients [26,27]. Specifically, in non-colorectal digestive cancers, the Panesage cohort revealed, through multivariate analysis, that a gait speed below 0.8 m/s is correlated with treatment adaptations compared to reference management [28]. Notably, the gait speed of 1.09 m/s in patients selected by oncologists to receive bi-chemotherapy for bronchial cancer exceeds the frailty threshold, indicating an intuitive consideration of this motor parameter by oncologists [29].

Gait disorders elevate the risk of falls and impede autonomy, requiring consideration in the selection of therapies, especially concerning chemotherapy neurotoxicity. Considering fall’s impact on morbidity and mortality, onco-geriatricians must identify sedentary lifestyles, analyse motor functions, and assess muscle mass. Comorbidities such as diabetes or a narrow lumbar canal, which could intensify the consequences of neurotoxicity, should be documented. Physical activity, even in older cancer patients, has been demonstrated to alleviate fatigue. In pancreatic cancer, physical activity not only enhances quality of life but also tends to improve overall survival [30].

#### 3.2.4. Cognitive Disorders

Cognitive disorders show an age-associated increase and are not consistently diagnosed, particularly in their early stages. These disorders may delay the diagnosis of cancer [31] and elevate mortality rates [32]. Although the period following a cancer diagnosis is not ideal for exhaustive cognitive testing, screening for pre-existing or cancer-related neurocognitive disorders is imperative due to their impact on cancer management. The Montreal Cognitive Assessment (MOCA) scale appears to be the most suitable tool for screening patients for cognitive disorders [33]. In the surgical context, memory disorders are a risk factor for postoperative confusion, which, in turn, is associated with postoperative complications, prolonged length of stay, and increased mortality [34,35,36]. Cognitive disorders also function as risk factors for poor chemotherapy tolerance.

Evaluating initial cognitive functioning is crucial, as certain cognitive issues are linked directly to cancer itself—through inflammation and fatigue—or indirectly through depressive symptoms. Various systemic treatments, whose specific impact on cognition is challenging to discern due to multiple influencing factors, also exhibit cognitive effects [37]. The concept of “chemobrain”, defining cognitive impairment associated with chemotherapy, has been extensively studied in adjuvant breast cancer, where 70% of patients report memory complaints during or after chemotherapy [38].

To date, there are no recommendations for adapting or selecting chemotherapies based on pre-existing cognitive impairment. A study involving patients with a median age of 77 awaiting first-line chemotherapy, including 42.9% with digestive cancer, revealed MMSE deterioration in 18.7% of patients. Factors associated with the risk of MMSE alteration during treatment included initial abnormal MMSE, altered Mini Nutritional Assessment (MNA), pain, and alteration of the QLQC30 quality of life questionnaire [39]. The cognitive functions affected by cancer treatments primarily involve memory, concentration, information processing speed, and executive functions. Cognitive disorders or memory complaints may evolve spontaneously after cancer treatment or persist over the long term, and cognitive rehabilitation can assist in managing them [40]. Studies investigating the effects of immunotherapy on cognitive status are also underway [41].

Finally, the detection of sensory disorders is essential to ensure proper understanding of shared information and contributes to mitigating postoperative delirium.

#### 3.2.5. Thymic Assessment

Depression is a common occurrence in oncogeriatrics, with a prevalence of 28.4% based on an assessment of over 1000 patients averaging 80 years old [42]. However, it is frequently under-diagnosed [43]. Several geriatric parameters, including polypharmacy, comorbidities, and functional status, are independently associated with the occurrence of depression. Notably, pancreatic cancer stands out among cancers with one of the highest rates of depression, estimated at around 40% [42].

#### 3.2.6. Impact of Comorbidities

Various scoring systems are available for assessing comorbidities, including the Charlson score (with different versions) and the CIRS-G. To standardize geriatric assessments, it is currently recommended to perform at least one assessment using the modified Charlson score [9]. When evaluating postoperative complications and causes of mortality in pancreatic and biliary surgery, impaired cardiorespiratory function and its management emerge as crucial factors.

After hepatic resection, mortality tends to be higher in older patients, primarily due to cardiorespiratory complications, as opposed to hepatic complications, which do not exhibit significant age-related differences [44]. Additionally, there is an increased risk of cardiorespiratory complications associated with pancreatic surgery [45,46].

#### 3.2.7. Therapeutics in Onco-Geriatrics: Limiting Polypharmacy, Iatrogenesis, and Drug Interactions

Age-related changes in body composition, as well as alterations in renal and hepatic function, modify the pharmacokinetics and risk of adverse effects of various molecules. Investigating potential drug interactions is imperative, given the frequent use of polypharmacy in this population. The prevalence of polypharmacy in the older patients varies widely, ranging from 13 to 92%, depending on the definition and characteristics of the studied population [47]. In the Panesage study, where polypharmacy is defined as taking ≥5 active ingredients per day, it was found to be 69% [27]. Collecting information about all the molecules taken by a patient necessitates specific interrogation, including inquiries about various prescriptions and self-medication practices, such as phytotherapy. The STOPP-START tool can aid in determining whether all prescribed medications are indicated or if any should be reconsidered [48].

Pharmaceutical reconciliation is advantageous when possible to limit iatrogenic effects resulting from drug interactions [49]. The impact of polypharmacy in older people with cancer on mortality is not clear but it is well established on morbidity [47]. Polypharmacy has been linked to a higher number of falls, drug interactions, and unplanned hospitalizations [50].

At the conclusion of the comprehensive geriatric assessment, clinicians obtain key elements to guide treatment decisions, but also to assess prognosis. Various geriatric parameters, such as the G8 score (prognostic of overall survival) [51], walking speed before therapeutic decision (predicting frailty with a threshold at 1 m/s) [27], and autonomy assessment by Activities of Daily Living (ADL) (predicting overall survival and postoperative complications) [10] offer valuable prognostic insights. Furthermore, scores combining oncological and geriatric parameters facilitate the estimation of short-term mortality risk, prompting considerations about the appropriateness of cancer-specific treatments when the risk is exceptionally high [52].

The geriatrician’s role involves continuous monitoring of the patient throughout their care journey, in collaboration with the organ specialist. Changes in geriatric parameters may necessitate specific adjustments in both geriatric and oncological interventions.

## 4. Support for Surgical Care

### 4.1. Preoperative Optimization

#### 4.1.1. Prognostic Evaluation of Surgical Risks Through Geriatric Assessment

Older adults and more comorbid patients face an elevated risk of complications during oncological surgery. The likelihood of experiencing a geriatric event (confusion, dehydration, falls, fractures, pressure sores) is correlated with the risk of specific complications, with higher occurrences observed in gastric or pancreatic surgeries compared to other abdominal locations [53].

#### 4.1.2. Prehabilitation

##### The Emergence of Prehabilitation

The importance of preoperative functional status in influencing postoperative recovery is now firmly established. It has been demonstrated that pre-therapeutic gait speed is a predictor of frailty [27]. In a study involving nearly 200 patients aged over 70 undergoing abdominal cancer surgery, superior pre-operative physical performance was associated with a reduced risk of severe complications and a lower rate of discharge to a rehabilitation ward compared to a return home [54]. Sarcopenic patients, as defined by the consensus of the European Working Group on Sarcopenia in Older People, are at a higher risk of postoperative complications and increased mortality following abdominal surgery [55].

These findings highlighted the need for preoperative interventions aimed at enhancing patients’ functional reserve, improving tolerance to the physiological stress of major surgery, and reducing the risks of postoperative complications, and give rise to the concept of prehabilitation [56].

##### What Type of Prehabilitation?

Prehabilitation typically consists of three components: physical exercise, nutritional support, and measures to reduce anxiety. While these components can be applied individually, a meta-analysis on patients with oesophagogastric cancer revealed that multimodal prehabilitation significantly reduced overall complication rates compared to standard care, unlike unimodal prehabilitation [57]. These findings align with broader research on abdominal and colorectal cancer surgeries [56,58,59].

The implementation of these three components remains highly heterogeneous and minimally validated, particularly for non-colorectal digestive cancers in older patients as most available data originate from studies on colorectal cancer or, more broadly, abdominal cancers.

The three components of prehabilitation are:

1. Adapted physical activity

Initiating prehabilitation requires an initial assessment, including estimation of the intensity of current physical activity in METs (Metabolic Equivalent of Task), and measurements of muscle mass and function. This assessment helps define a personalized program with specific improvement objectives.

The physical activity component typically includes three approaches, which may be used individually or in combination: Aerobic Exercises (AE), Resistance Training (RT), and Inspiratory Muscle Training (IMT). Before major abdominal surgery, a potential clinical advantage is observed when combining AE and IMT [60].

The optimal frequency, intensity, and modalities (autonomous, partially supervised, or fully supervised) remains unknown. Professional supervision, when feasible, may enhance the positive effects of exercise [61] but the optimal frequency, intensity, and modalities (autonomous, partially supervised, or fully supervised) remains unknown.

2. Nutritional management

Nutritional management aims to prevent or address undernutrition and promote anabolism. This primarily involves a high-protein, high-calorie diet with food fortification and oral nutritional supplements. The various options for taking oral nutritional supplements must be explained to ensure patient compliance. Enteral nutrition is preferred if oral intake is insufficient, as it prepares the digestive tract for resumption of feeding and is less prone to complications other than inhalation pneumonitis. In cases of cognitive impairment or risk of inhalation, artificial nutrition may not be recommended. If the digestive tract is non-functional, or if enteral nutrition fails, parenteral nutrition is recommended. The European Society for Clinical Nutrition and Metabolism recommends perioperative immuno-nutrition for patients scheduled for upper gastrointestinal cancer surgery to reduce the risk of postoperative infection and length of stay [62].

3. Psychological care

Anxiety and depression are prevalent among cancer patients, especially as age advances. Symptoms of depression in older patients can be less apparent than in younger patients but have a negative impact on post-operative follow-up, including length of stay, recovery, pain, and quality of life. Psychological care is therefore essential and should be considered alongside physical and nutritional status [63]. Psychological management can also serve as motivational support for patients undergoing lifestyle changes. Despite this, few studies focus on the psychological component of prehabilitation. A 2015 review of psychological prehabilitation before cancer surgery indicates that it improves quality of life and physical symptoms, with some studies showing sustained benefits at 1-year post-surgery. Some studies even suggest that psychological care may enhance immune function [64]. More recently, a 2022 literature review showed a trend towards improved psychological outcomes following psychological prehabilitation, particularly when psychologist-led [65].

##### Prehabilitation Duration

The optimal duration of prehabilitation appears to be 3 to 6 weeks, ideally integrated with necessary treatments and examinations prior to surgery [66,67], yet no specific data on non-colorectal digestive cancer surgery are available.

##### Population and Efficacy of Prehabilitation

The effectiveness of prehabilitation, particularly concerning traditional surgical criteria, has yet to be demonstrated, possibly due to study and evaluation criteria heterogeneity. In colorectal cancer, despite numerous studies, no improvement in survival has been proven [68]. This may be partly linked to the choice of target population. The article by M. Coca Martinez and F. Carli underscores that studies concentrating on high-risk, frail, or older patients are more likely to demonstrate improvements in complication rates or length of stay, in contrast to those involving a broader patient population [58]. Also, in colorectal cancer, patients with a walking distance of less than 400 m in the 6-min test improve their walking distance more and are more likely to recover their baseline abilities postoperatively than those with better preoperative walking abilities [69], suggesting that prehabilitation yields greater potential for recovery in frail subjects.

On the positive side, evidence suggests that prehabilitation enhances both pre- and post-operative physical conditions and reduces the length of hospital stay. For patients specifically managed for carcinologic oesophagogastric surgery, improvements in functional abilities have been observed after prehabilitation [70]. Another study in a similar population demonstrates a reduction in postoperative pneumonitis and morbidity, assessed by complications greater than or equal to II on the Clavien Dindo scale, along with improvements in length of stay and quality of life for dyspnoea and physical functioning [71]. In a meta-analysis covering all digestive cancers, prehabilitation was associated with a reduction in length of stay by 1.78 days, although it showed no demonstrated effect on functional capacity, complications, or postoperative mortality [72].

Contrastingly, a 2022 review focusing on hepato-pancreato-biliary surgery did not find the reduction in length of stay associated with prehabilitation [73]. In a specific study on pancreatic cancer by Ngo-Huang, aiming for mainly physical prehabilitation during neoadjuvant treatment, improvements in physical capacities were observed in the intervention arm. However, no reduction in complications or postoperative length of stay was found, possibly due to a lack of statistical power and a relatively younger patient population (average age 66) [74]. In their 2022 study, Deprato et al. found a significant improvement in preoperative and postoperative physical performance while length of stay, complication rates, and mortality demonstrated no statistically significant differences, but trended towards benefit from prehabilitation [75].

There is not enough evidence yet to support the systematic inclusion of prehabilitation in ERAS protocols for non-colorectal digestive cancer surgeries. However, it could provide a valuable addition to conventional protocols, particularly for frail geriatric patients undergoing major surgeries or neoadjuvant treatments [76].

In addition to the tri-modal prehabilitation approach outlined earlier, it remains crucial to uphold classic peri-operative management practices. This includes endeavours such as smoking and alcohol cessation where possible, meticulous management of co-morbidities, and vigilant prevention of anaemia. In the targeted population, special attention should be dedicated to the prevention of confusion, ensuring a comprehensive approach to optimize patient outcomes throughout the surgical journey.

### 4.2. Post-Operative Co-Management

#### 4.2.1. Co-Management Between Surgeons and Geriatricians: Impact on Mortality and Surgical Complications

The study conducted by Armin Shahrokni et al., encompassing over 30% of patients with digestive cancers, half of which were non-colorectal, revealed significant findings related to co-management between surgeons and geriatricians. The co-management approach demonstrated a notable reduction in mortality at 3 months, with an odds ratio (OR) of 0.58. However, despite the positive impact on mortality, there was no observed difference in surgical complications [77]. This underscores the importance of further exploration into the specific aspects of comorbidity management within the co-management framework. Several prospective interventional studies on the benefits of co-management between surgeons and geriatricians are underway.

#### 4.2.2. Fast-Track Surgery

The post-operative management of older patients should align with modern concepts such as “Fast-Track Surgery” or Enhanced Recovery After Surgery (ERAS) protocols. This approach involves a multimodal, multidisciplinary strategy to control the response to surgical stress and alleviate its consequences, resulting in accelerated post-operative recovery and reduced medical consultations [78,79,80].

The key principles of Fast-Track Surgery include minimizing aggression (utilizing minimally invasive approaches, avoiding unnecessary interventions such as drainage, urinary, or gastric catheterization), early refeeding and mobilization (getting up and into the chair on day 0 or 1), and sparing the use of morphine for postoperative analgesia. Recent evidence supports the applicability of Fast-Track Surgery to older patients, and shows and shorter length for older adults who benefited from ERAS [81,82].

ERAS even demonstrates a considerable reduction in the postoperative complication rates for studies interested in colorectal cancer [83,84,85,86], but evidences are poorer for non-colorectal cancers. However, a study on gastric cancer found that ERAS patients had a shorter hospital stay after surgery and fewer Clavien-Dindo grade IIIa complications than those in the conventional group [87].

This approach is validated for general populations undergoing digestive surgery and is applicable to older patients, provided the nursing teams are equipped to handle the specificities of this demographic [88,89].

#### 4.2.3. Post-Operative Delirium: Prevention and Management

The incidence of postoperative delirium is reported to be 15% for controlled surgeries and 20% for emergency procedures. These disorders complicate healthcare team management, increase postoperative mortality (19% vs. 8%), and extend the length of stay (21 days vs. 8 days) [90]. Confounding risk factors include high age, high American Society of Anaesthesiologists (ASA) score, low body mass index (BMI), low albuminemia, intraoperative hypotension, intraoperative blood transfusions, and a history of excessive alcohol consumption [91].

A systematic search for confusion syndrome, which can be efficiently and promptly conducted using the Confusion Assessment Method [92], enables early management if post-operative delirium syndrome is confirmed.

The most effective strategy involves the prevention of delirium, primarily relying on a multidimensional, non-pharmacological approach as a first-line treatment. These preventive measures, which also serve as initial interventions in the presence of confirmed confusion, encompass various aspects [93,94], including:

- Detecting pre-existing cognitive disorders.
- Limiting iatrogenicity: this involves restricting the prescription of certain medications that may contribute to confusion, such as anticholinergic drugs, certain painkillers, such as tramadol or meperidine, and psychotropic drugs.
- Ensuring proper correction of any sensory impairments and reorientation.
- Addressing sleep disorders, including sleep apnoea syndromes.
- The other three points overlap with ERAS: Managing transit or urinary disorders, encouraging early postoperative walking and providing technical aids if necessary, and managing pain.

Numerous studies come to the conclusion that postoperative management of the older adults, including ERAS, is associated with a reduced rate of confusion and an improved preservation of cognitive function [95,96].

When non-medication approaches prove insufficient to manage postoperative confusion syndrome, cautious medication management may be considered. This is particularly relevant in cases where confusion hinders essential treatments or examinations or generates considerable distress. In such instances, the following principles should guide medication use:

1. Prefer the use of a single medication, chosen based on the specific symptoms of the patient.
2. Administer the medication at the lowest effective dose.
3. Prefer oral administration whenever possible.
4. Choose medications with the least anticholinergic effects possible to mitigate potential cognitive side effects.

It is important to be cautious in medication management and prioritize the well-being of the older patient. If benzodiazepines are deemed necessary (for anxiety or alcohol withdrawal syndrome), opting for short-acting formulations is preferable. In cases of severe agitation that may lead to harm to self or others, low-dose neuroleptic treatment may be considered. However, such medication usage should be carefully monitored and reassessed early in the treatment process. Melatonin can be an option for circadian rhythm disturbances [97].

It is noteworthy that while pharmacological interventions may be utilized to manage acute symptoms, no pharmacological treatment has been conclusively proven effective in preventing or treating postoperative confusional syndrome [98]. The decision to use medications should be made on a case-by-case basis, weighing the potential benefits against the risks and considering the specific circumstances of the patient. Additionally, ongoing assessment and re-evaluation are crucial elements in the pharmacological management of postoperative confusion syndrome.

**Figure 1:**
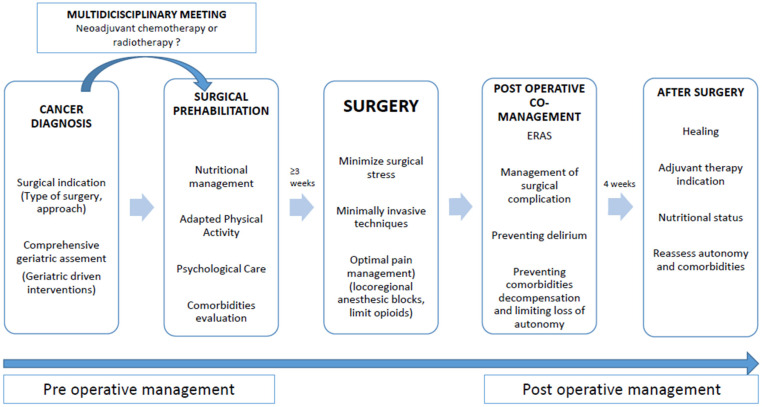
Optimal perioperative management of older patients.

Support for surgical care and co-management between surgeon and geriatrician are summarized in Figure 1.

## 5. Support for Systemic Treatments Management

### 5.1. Toxicity of Systemic Treatments in Older Patients with Digestive Cancers

Predicting chemotherapy toxicity in older individuals poses a significant challenge. Two specific predictive scores designed for older patients, namely the Chemotherapy Risk Assessment Scale for High-Age Patients (CARG) and the Chemotherapy Risk Assessment Scale for High-Age Patients (CRASH) score, have been developed [99,100]. These scores incorporate both geriatric and oncological criteria to assess the risk of chemotherapy-related toxicity in older individuals. While it has not been specifically studied in the context of digestive cancers, no difference in efficacy between these two scales has been found [101].

Immunotherapy also presents challenges in this population. Although studies across diverse cancers have reported similar toxicity profiles for immunotherapy in older and younger patients, there are higher rates of treatment discontinuation in older individuals experiencing the same level of toxicity [102,103]. To our knowledge, there are no specific studies examining the toxicity of immunotherapy in older individuals with digestive cancers.

In view of the digestive underlying neoplasia, and the reduced absorption of vitamins with age, we recommend that vitamins B9 and 12 be measured and supplemented if necessary, and that iron be supplemented by venous route if necessary, prior to chemotherapy or immunotherapy.

Similarly, as the action is muscular and not just bone-related, pre-therapeutic vitamin D dosage and supplementation may be useful [104].

Geriatric specificities related to the tolerance of systemic treatments in digestive oncology are presented in Table 1

### 5.2. Co-Management Between Oncologists and Geriatricians

Demonstrating the benefits of co-management between oncologists and geriatricians poses challenges due to diverse care pathways and numerous inherent evaluation biases. Nevertheless, findings from several recent studies indicate a reduction in treatment toxicity and improved completion of systemic treatments attributed to geriatric management. It is noteworthy that these studies are not exclusive to non-colorectal digestive cancers [105].

For instance, in the GAIN study, interventions based on comprehensive geriatric assessment (CGA) resulted in a decreased incidence of grade III toxicity at 50.5% compared to 60.3% in the control group (*p* = 0.02) [106]. While there was an increase in the formulation of advance directives, no significant differences were observed in emergency room visits, unscheduled hospitalizations, length of stay, survival, chemotherapy duration, or dose. A recent literature review reported a favourable treatment completion rate in 4 out of 6 studies following geriatric assessment [107]. Similarly, the GAP 70 study demonstrated a reduction in grade 3–5 toxicities (Hazard Ratio of 0.74), accompanied by geriatric benefits such as fewer falls and lower discontinuation rates in the intervention arm [108]. The INTEGERATE study corroborated these findings by revealing an enhancement in quality of life and a decrease in unscheduled hospitalizations, aligning with similar results from other studies [107,109].

However, the impact of geriatric management on overall survival with systemic treatments remains uncertain, with conflicting results across studies. In a recent “before/after” study involving 40% of oesophagogastric cancers within our target population, a positive gain in survival was observed following the implementation of geriatric management [110].

## 6. Conclusions

Limited geriatric-specific data exist for non-colorectal digestive cancers in current literature. Nevertheless, the merits of incorporating geriatric management into the collaborative efforts of surgeons and oncologists are gaining recognition and are increasingly being validated, as confirmed by the review of oesophagogastric cancers by T. Aparicio et al. [111]. While the impact on mortality remains challenging to establish due to various biases, there is accumulating evidence suggesting positive outcomes in terms of criteria such as decreased chemotherapy complications and shorter surgical stays.

It is essential to emphasize that the role of the geriatrician is complementary to that of other specialists. Their involvement spans decision-making processes, care pathway management, and the prevention of comorbidity decompensation, geriatric syndromes, and loss of autonomy. This interdisciplinary approach aims to address the unique needs and challenges faced by older patients with non-colorectal digestive cancers, enhancing overall patient care and outcomes. Further research and ongoing studies are expected to provide additional clarity and insights into the specific benefits of geriatric management in this context.

## Figures and Tables

**Table 1:** Geriatric specificities related to the tolerance of systemic treatments in digestive oncology.

Molecules	Main Toxicities	Comorbidities to Look For	Geriatric Outcomes	Recommended Course of Action
**OXALIPLATINE**	Neuropathic	Diabetes, narrow lumbar canal, other causes of neuropathy	Increased falls, functional decline, need for assistance with dressing and grooming	↓ dose, stop
			Infections	Home aids, remote alarm, physiotherapy, technical aids
	Hematologic	Hemopathy, low bone marrow reserves	Anaemia: falls, cardiac decompensation, loss of autonomy	↓ dose
			Infections, falls, wounds	Influenza/COVID/pneumococcal vaccination
				Erythropoietin
	Digestive	Nutrition		Weight monitoring, dietary management, caregiver education
**5-FLUOROURACIL**	Coronary spasm	Severe unstable coronary artery disease		Cardiologic advice
	Transit disorders	Incontinence, gait disorders	Risk of falls, undernutrition	↓ dose, ↓ bolus
			Risk of dehydration	Perforated chair, protection, hydration, dietary care
	Hand-foot syndrome	Gait disorders	Risk of falls, loss of autonomy (dressing, cooking)	↓ dose
				Implementation of aids
**TAXANES**	Neuropathic	Diabetes, narrow lumbar canal, other causes of neuropathy	Increased falls, functional decline, need for assistance with dressing and grooming	↓ dose, stop
				Home aids, remote alarm, physiotherapy, technical aids
**BEVACIZUMAB**	High blood pressure	High blood pressure, heart failure	Risk of decompensation of cardiovascular comorbidities	Monitoring of blood pressure and weight
	Proteinuria	Renal insufficiency		
	Thrombosis and haemorrhage	History of thrombosis, anticoagulant, valve prosthesis		Discuss low-molecular-weight heparin LMWH if direct oral anticoagulant (DAO)or Vitamin K antagonist VKA
	Healing disorders	Unscheduled surgery for comorbidity		Cancel or differ scheduled non-urgent surgery
**IRINOTECAN**	Transit disorders	Incontinence, gait disorders	Increased falls	↓ dose, stop
			Risk of dehydration and malnutrition	Wardrobe chair, diapers, incontinence briefs, hydration, dietary management
	Medullary	Hemopathy, low bone marrow reserves	Infections	Influenza/COVID/pneumococcal vaccination
			Anaemia: falls, cardiac decompensation, loss of autonomy	Erythropoietin
				Providing Human assistance
**NIVOLUMAB**	Asthenia	General condition	Loss of autonomy	Adaptation of aids
**PEMBROLIZUMAB**	Rash	Xerosis, pruritus	Pruritus, wounds	Emollient, antihistamines (avoid hydroxizine-type anticholinergics due to cognitive and urinary risk)
	Diarrhoea	Incontinence, walking disorders	Increasing falls, risk of dehydration	Remote alarm, therapeutic education for patient or caregiver
			Walking difficulties	
	Arthralgia	Osteoarthritis, polymyalgia rheumatica, rheumatoid arthritis		Analgesia, rheumatologic advice, technical aids, remote alarm
	Thyroid dysfunction	Cognitive impairment	Increased cognitive impairment, heart disease	Endocrinologist advice
**ATEZOLIZUMAB**	Asthenia	Cardiopathy	Loss of autonomy	See above
	Pruritus			
	Arthralgia			
	Nausea			

## Data Availability

Not applicable.

## Large Language Model Statement

As the authors are not native British speakers, they utilized ChatGPT 4.0 model as an editing tool to: review and amend grammatical and spelling mistakes; ensure linguistic consistency and coherence. Moreover: to ensure that ChatGPT 4.0 did not introduce plagiarized wording while correcting grammatical mistakes or linguistic inconsistencies, the manuscript has been submitted to two different plagiarism checkers: duplichecker.com assessed on the 2 June 2024 which found 99% unique text and originality.ai which found 100% unique text.
